# Next Generation Phenotyping Using the Unified Medical Language System

**DOI:** 10.2196/medinform.3172

**Published:** 2014-03-18

**Authors:** Tomasz Adamusiak, Naoki Shimoyama, Mary Shimoyama

**Affiliations:** ^1^Human and Molecular Genetics CenterMedical College of WisconsinMilwaukee, WIUnited States; ^2^Department of SurgeryMedical College of WisconsinMilwaukee, WIUnited States

**Keywords:** meaningful use, semantic interoperability, UMLS, SNOMED CT, LOINC, RxNorm, CPT, HCPCS, ICD-9, ICD-10

## Abstract

**Background:**

Structured information within patient medical records represents a largely untapped treasure trove of research data. In the United States, privacy issues notwithstanding, this has recently become more accessible thanks to the increasing adoption of electronic health records (EHR) and health care data standards fueled by the Meaningful Use legislation. The other side of the coin is that it is now becoming increasingly more difficult to navigate the profusion of many disparate clinical terminology standards, which often span millions of concepts.

**Objective:**

The objective of our study was to develop a methodology for integrating large amounts of structured clinical information that is both terminology agnostic and able to capture heterogeneous clinical phenotypes including problems, procedures, medications, and clinical results (such as laboratory tests and clinical observations). In this context, we define phenotyping as the extraction of all clinically relevant features contained in the EHR.

**Methods:**

The scope of the project was framed by the Common Meaningful Use (MU) Dataset terminology standards; the Systematized Nomenclature of Medicine Clinical Terms (SNOMED CT), RxNorm, the Logical Observation Identifiers Names and Codes (LOINC), the Current Procedural Terminology (CPT), the Health care Common Procedure Coding System (HCPCS), the International Classification of Diseases Ninth Revision Clinical Modification (ICD-9-CM), and the International Classification of Diseases Tenth Revision Clinical Modification (ICD-10-CM). The Unified Medical Language System (UMLS) was used as a mapping layer among the MU ontologies. An extract, load, and transform approach separated original annotations in the EHR from the mapping process and allowed for continuous updates as the terminologies were updated. Additionally, we integrated all terminologies into a single UMLS derived ontology and further optimized it to make the relatively large concept graph manageable.

**Results:**

The initial evaluation was performed with simulated data from the Clinical Avatars project using 100,000 virtual patients undergoing a 90 day, genotype guided, warfarin dosing protocol. This dataset was annotated with standard MU terminologies, loaded, and transformed using the UMLS. We have deployed this methodology to scale in our in-house analytics platform using structured EHR data for 7931 patients (12 million clinical observations) treated at the Froedtert Hospital. A demonstration limited to Clinical Avatars data is available on the Internet using the credentials user “jmirdemo” and password “jmirdemo”.

**Conclusions:**

Despite its inherent complexity, the UMLS can serve as an effective interface terminology for many of the clinical data standards currently used in the health care domain.

## Introduction

### The Definition of Meaningful Use

The Health Information Technology for Economic and Clinical Health Act, enacted as part of the American Recovery and Reinvestment Act of 2009, introduced the concept of Meaningful Use (MU) of information technology in health care. The definition of MU in this context is complex and consists of several objectives and measures that health care providers have to demonstrate in three stages and within strict timelines in order to be eligible for early adopter incentives, and later on to avoid penalties for noncompliance starting in 2015. The MU legislation was designed to transform US health care through the development of processes and standards to capitalize on information in individual medical records and to create data resources that would result in better health care for the greater population.

As part of this process, the legislation mandated the use of standard terminologies for the electronic exchange of health information. In particular, the Office of the National Coordinator for Health Information Technology defined a common set of MU data elements for which certification would be required across a number of electronic health records (EHR) interoperability certification criteria. The EHR interoperability can be further categorized into: (1) foundational, the ability to send information from one system to another, but without the need for interpretation on the receiving end; (2) structural, the syntax, format of simply messaging standards to provide transport of the information; and finally, the most challenging, (3) semantic interoperability, which allows the receiving system to interpret and integrate the received information [1]. The Common MU Dataset has profound consequences for semantic interoperability, as it defines a set of strict terminology standards to be used within a certified EHR. A summary of these is provided in Table 1 and introduced in more detail below.

### Biomedical Terminologies

The Systematized Nomenclature of Medicine, Clinical Terms (SNOMED CT) is one of the most widely used biomedical terminologies in the world. It provides terms, synonyms, and relations covering a number of clinical domains including diseases, findings, and procedures [2]. The Logical Observation Identifiers Names and Codes (LOINC) is a universal standard for identifying laboratory observations. It is considered the *lingua franca* of the clinical observation exchange with its more than 20,000 users in 150 countries [3]. The National Drug Code (NDC) is a well established drug standard that is required in electronic pharmacy claims [4]. The RxNorm is a more recent standardized drug nomenclature designed to facilitate medication reconciliation. It incorporates a number of other drug terminologies, as well as maps to the NDC [5]. The Health care Common Procedure Coding System (HCPCS), maintained by the Centers for Medicare & Medicaid Services (CMS), is a standardized coding system for describing items and services provided in the delivery of health care [6]. It incorporates the Current Procedural Terminology (CPT), a coding system maintained by the American Medical Association, to identify medical services and procedures used by physicians and other health care professionals [7]. The American Dental Association, for accurate reporting of dental treatment [8], developed the Code on Dental Procedures and Nomenclature (CDT). 3M Health Information Systems have developed the International Classification of Diseases Tenth Revision Procedure Coding System (ICD-10-PCS) for the CMS as a replacement for the International Classification of Diseases Ninth Revision Clinical Modification (ICD-9-CM) [9]. The International Classification of Diseases Tenth Revision Clinical Modification (ICD-10-CM) does not contain a procedure classification in contrast to its predecessor ICD-9-CM, and this is where ICD 10 PCS complements ICD-10-CM. The HCPCS, CDT, and ICD-9-CM are used in US electronic transaction claims with planned replacement of the ICD-9-CM by the ICD-10 in October 2014.

US health care relies on a number of different clinical terminology standards with varying levels of overlap and maturity. This already intricate landscape is further complicated by the disparity between billing and MU reporting. For example, SNOMED CT is not allowed in claims reporting and RxNorm combines multiple NDCs under one substance code, rendering detailed package and labeler based billing difficult. The clinical informatics community is now recognizing the need for new tools capable of consuming these heterogeneous resources, hence the term “next generation phenotyping” [10]. In this context, phenotyping is defined as extracting all clinically relevant information from raw EHR data. These clinically relevant features include problems, procedures, medications, and clinical results (such as laboratory tests and clinical observations) annotated with standard clinical terminologies.

**Table 1:** Common MU Dataset defined in Stage 2 MU Final Rule (Federal Register Vol. 77, No. 171, September 4, 2012) and corresponding vocabulary standards.

Common MU Dataset	Vocabulary standard
1. Patient name	N/A
2. Sex	N/A
3. Date of birth	N/A
4. Race	The OMB^a^ Standards for Maintaining, Collecting, and Presenting Federal Data on Race and Ethnicity, Statistical Policy Directive No. 15, as revised, October 30, 1997
5. Ethnicity	OMB
6. Preferred language	As specified by the Library of Congress, ISO^b^639-2 alpha-3 codes limited to those that also have a corresponding alpha-2 code in ISO 639-1
7. Smoking status	Any of the following SNOMED CT^c^ codes- (1) Current every day smoker, 449868002 (2) Current some day smoker, 428041000124106 (3) Former smoker, 8517006 (4) Never smoker, 266919005 (5) Smoker, current status unknown, 77176002 (6) Unknown if ever smoked, 266927001 (7) Heavy tobacco smoker, 428071000124103 (8) Light tobacco smoker, 428061000124105
8. Problems	At a minimum, SNOMED CT International Release July 2012 and US Extension to SNOMED CT March 2012 Release
9. Medications	RxNorm, August 6, 2012 Release
10. Medication allergies	RxNorm, August 6, 2012 Release
11. Laboratory tests	LOINC^d^ version 2.40
12. Laboratory values/results	N/A
13. Vital signs (height, weight, BP^e^, BMI^f^)	N/A
14. Care plan fields including goals and instructions	N/A
15. Procedures	At a minimum, SNOMED CT International Release, July 2012 with US Extension to SNOMED CT March 2012 or the combination of HCPCS^g^ and CPT^h^ 4 Optional, CDT^i^, ICD-10-PCS^j^
16. Care team members	N/A

^a^OMB=Office of Management and Budget

^b^ISO=International Organization for Standardization

^c^SNOMED CT=Systematized Nomenclature of Medicine, Clinical Terms

^d^LOINC=Logical Observation Identifiers Names and Codes

^e^BP=blood pressure

^f^BMI=body mass index

^g^HCPCS=Health care Common Procedure Coding System

^h^CPT=Current Procedural Terminology

^i^CDT=Code on Dental Procedures and Nomenclature

^j^ICD-10-PCS=International Classification of Diseases, Tenth Revision, Procedure Coding System

### Local Coding Systems

Many organizations develop their own local coding systems to address these challenges. In fact, to meet the 2014 Edition EHR Certification Criteria, providers are not required to use terminology standards internally as long as they are able to consume them for data portability and clinical quality measures reporting. Convergent Medical Terminology (CMT) is an example of such a solution developed by Kaiser Permanente (KP). CMT serves as the common terminology across all of the KP enterprise, and, at its core is comprised of SNOMED CT, LOINC, and First DataBank drug terminology [11]. However, local coding systems require considerable resources to develop and maintain, and, *ipso facto,* add another layer of complexity to an already convoluted process.

We therefore propose a different solution that relies on the Unified Medical Language System (UMLS) developed and maintained by the National Library of Medicine (NLM) [12]. All of the aforementioned terminology standards are already integrated within the UMLS, which incorporates more than a hundred vocabularies in the biomedical domain. Additionally, the UMLS provides a consistent categorization of all concepts represented in the UMLS Metathesaurus within the UMLS Semantic Network. This makes it an ideal candidate for clinical data integration. While the UMLS has not been designed with a specific intent for bioinformatics, it also incorporates many of the bioinformatics resources, such as the Gene Ontology, the Medical Subject Headings, and the Online Mendelian Inheritance in Man (OMIM), which can further facilitate translational research by bridging clinical informatics and bioinformatics [13].

### Significance of This Study

There is now a significant need for integrating patient data from multiple sources, as well as supporting ontology driven querying and reporting on a large scale basis as the transformation of health care from paper to electronic progresses. The UMLS has been widely used as a terminology repository [13,14], in ontology related research [15,16], text mining (via MetaMap) [17], and text processing applications [18]. To our knowledge, with the exception of one proof-of-concept study [19], it has never been actually integrated directly into a clinical workflow as a terminology standard itself. The reasons for this are twofold: (1) the UMLS is technically challenging to work with due to its sheer size and complexity. It encompasses almost three million clinical concepts and eight million synonyms connected by almost 35 million relations (2013AB version). The hardware capabilities to work with such massive terminologies have only recently become available. And (2) before MU, there has been little terminology standardization in the EHR that would warrant an effort to integrate multiple vocabularies. To this day, with the exception of the rather limited coding of insurance claims, many hospital systems still use local coding schemes, which require cumbersome manual translation.

## Methods

### Data Model

Lightweight object models can rely on ontologies instead of modeling semantics explicitly. We have previously demonstrated this approach in the Observ-OM and VarioML models that were specifically validated for phenotype and genotype information by the GEN2PHEN [20] Consortium [21,22]. At its core, Observ-OM uses only four basic concepts to represent any kind of observation: (1) target, (2) feature, (3) protocol, and (4) value. To this effect, patients become simply collections of observations annotated with clinical terminologies. Each observation has at least one ontology term attached. Overcoding, for example, attaching multiple semantically similar concepts from different vocabularies to a single clinical observation, facilitates information retrieval when code similarity or equivalence have not yet been established in the UMLS. “Hemoglobin; glycosylated (A1C)” (CPT:83036) and “Glucohemoglobin measurement” (SNOMEDCT:40402000) are examples of two such concepts. Multiple terms can also provide additional context, for example, the method used to observe a phenotype (typically with LOINC), while keeping the data model flexible.

Additional semantic information can be derived from the semantic type of the UMLS concept used in the annotation. For example, the concept of Warfarin is typed in the UMLS Semantic Network as a Pharmacologic Substance. Thus, any observation about Warfarin can be inferred to be a medication for the purpose of querying or reporting. Where this is insufficient, we used explicit value sets. For example, the Common MU Dataset defines a set of SNOMED CT terms that together comprise smoking status (see Table 1). In this respect, we also created a custom value set based on the Office of Management and Budget (OMB) standard to represent ethnicity (see Table 2).

**Table 2:** Clinical Avatars data mapped to the UMLS via MU ontologies.

Clinical Avatars			MU source mapping (direct)	UMLS mapping (automatic)	Term label
**Gender**			None		
	F			C0015780	Female
	M			C0024554	Male gender
**Race**			OMB standard		
	African American			C0085756	African American
	Native American			C1515945	American Indian or Alaska Native
	Asian			C0078988	Asians
	White			C0043157	Caucasians
	(no data)			C0086409	Hispanic or Latino
	Pacific Islander			C1513907	Native Hawaiian or other Pacific Islander
	Other/unknown			C1532697	Unknown racial group
Height			LOINC:3137-7	C0365282	Body height measured
Weight			LOINC:3141-9	C0365286	Body weight measured
BSA^a^			LOINC:3139-3	C0365285	Body surface area measured
INR^b^			LOINC:34714-6	C1369580	INR in blood by coagulation assay value
**Smoker**					
		Y	SNOMED CT:77176002	C0337664	Smoker
		N	SNOMED CT:8392000	C0337672	Nonsmoker
**DVT** ^c^					
		Y	SNOMED CT:128053003	C0149871	Deep venous thrombosis
		N	SNOMED CT:413076004	C1446197	No past history of venous thrombosis
**AMI** ^d^					
		Y	SNOMED CT:57054005	C0155626	Acute myocardial infarction
		N	SNOMED CT:301121007	C0577811	Myocardial perfusion normal
CYP2C9			LNC:46724-1	C1830800	cyp2c9 gene mutations found [identifier] in blood or tissue by molecular genetics method nominal
CYP2C92			LNC:56164-7	C2734139	cyp2c9 gene allele 2 [identifier] in blood by molecular genetics method nominal
CYP2C93			LNC:56165-4	C2734141	cyp2c9 gene allele 3 [identifier] in blood by molecular genetics method nominal
VKORC1			LNC:50722-8	C1978717	vkorc1 gene mutations found [identifier] in blood or tissue by molecular genetics method nominal
VKORC1A			LNC:50722-8	C1978717	vkorc1 gene mutations found [identifier] in blood or tissue by molecular genetics method nominal
VKORC1G			LNC:50722-8	C1978717	vkorc1 gene mutations found [identifier] in blood or tissue by molecular genetics method nominal
Warfarin			RxNorm:11289	C0043031	Warfarin

^a^BSA=body surface area

^b^INR=international normalized ratio

^c^DVT=deep vein thrombosis

^d^AMI=acute myocardial infarction

### Terminology Server

The terminology service is built on top of a local database, which is populated with a standard set of vocabularies in the UMLS Active Release (subset of the full release, which includes only the actively updated terminologies). The UMLS is loaded into an Oracle database 11g using the Structured Query Language (SQL) scripts provided with the UMLS distribution and updated in sync with its semiannual release cycle. The currently loaded version is displayed dynamically in the scorecard section of the project home page. As a reference, a 2013AB version set incorporating 89 UMLS terminologies included 2,805,252 unique concepts and 8,622,812 synonyms. RxNorm information is loaded as part of the UMLS distribution, rather than through its own separate release.

**Figure 1:**
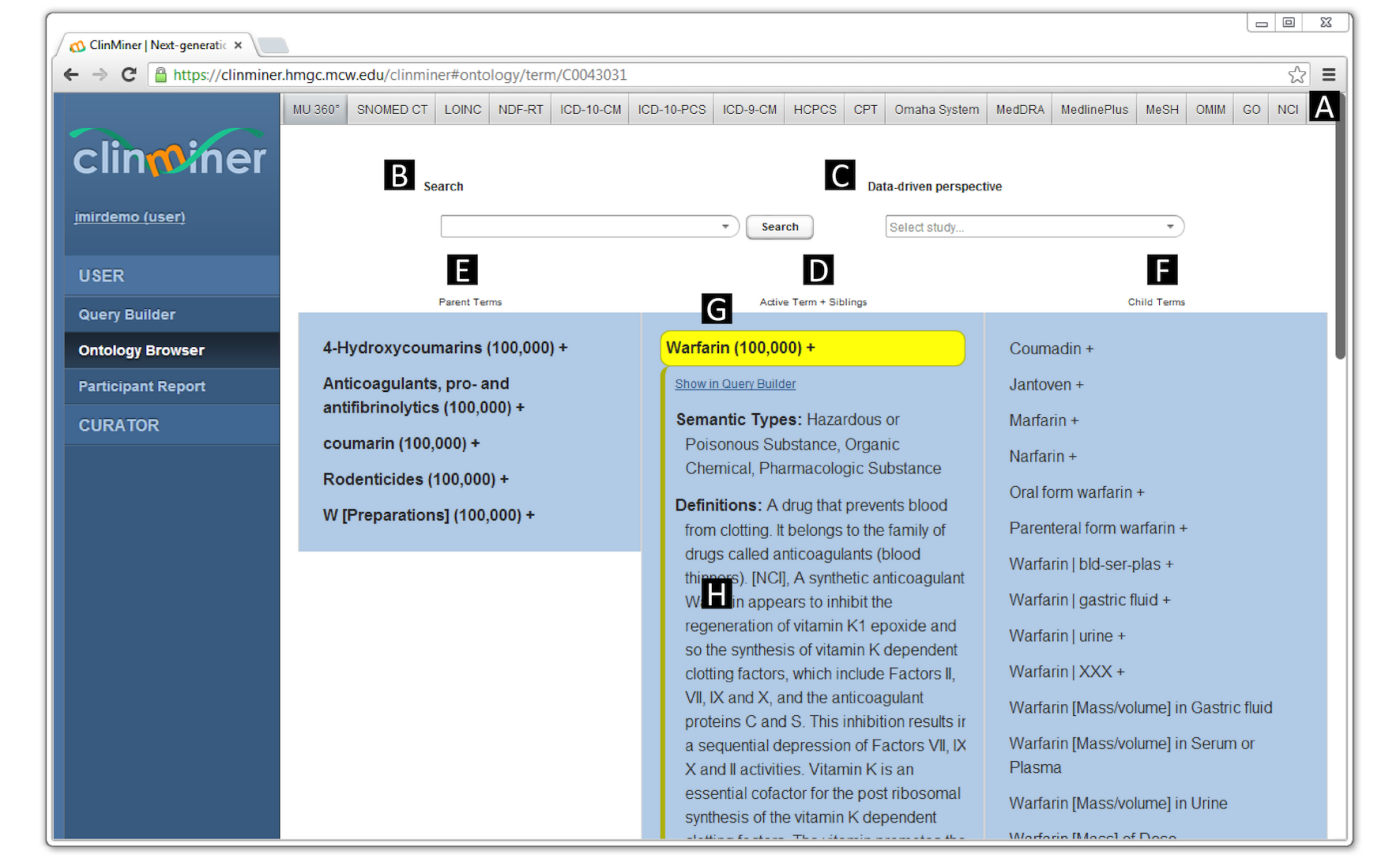
Screenshot of ClinMiner’s integrated terminology browser. The tabs allow switching between different terminologies and the integrated MU 360 view, default choice (A). Searching. Typing a query into the input field (B) brings up autosuggestions. Selecting a particular string populates the middle panel (D) with search results. Selecting a search result brings back the hierarchical view with the selected term (Warfarin) highlighted in yellow (G). Browsing. Parents of the active term are displayed in the left pane and child terms are displayed in the right pane (F). Meta data for the active term including semantic types, definitions, and non-isa relations to other concepts are displayed in a vignette directly below (H). A plus sign (+) after the term label denotes concepts with children, and the number in brackets reflects the number of participants annotated to a particular term (or its children) in the database. Selecting a study from the drop-down list (C) enables the data driven perspective that displays a compact terminology tree limited to only relevant concepts.

For the sake of usability, only a preconfigured subset is displayed as navigable tabs at the top of the browser window (Figure 1 shows this at the top right of the figure). However, all the sources included in the UMLS are potentially browsable and are used in synonym expansion.

The UMLS comes preconfigured with a broad set of indexes that optimize querying. We use one additional index on top of the attribute value column in the attribute table (MRSAT.ATV) to optimize a dedicated NDC search, which does not exist otherwise as a term code in the concept table (MRCONSO). A label and synonym search was implemented using Oracle Text, a set of Oracle based tools for building text query and document classification applications that provides indexing and text classification capabilities. Individual text tokens are indexed using the term frequency, inverse of the document frequency algorithm, reflecting how often a particular string occurs in the UMLS [23].

Rather than using a complex advanced search interface, we have a single search box that relies on the query relaxation approach (Figure 1). Depending on the context (eg, NDC search requires a different algorithm), the original user query is expanded on the database side into progressively more relaxed versions of the original query. Every search sequence starts with an exact phrase match; then progresses into matching all the tokens in a close proximity (NEAR Procedural Language/Structured Query Language operator); then all words matched (AND) in a phrase; then most words matched (ACCUMulate); and finally falls back to stemming, fuzzy matching and wildcard expansion.

Interpreting a query string using different operator combinations simultaneously allows for a more concise query design. For example, if a user enters a query “rash on examination”, the application can interpret the query in parallel as a single phrase *“*rash on examination*”* and “rash*” OR “*on*” OR “*examination*”* to increase recall. Fuzzy and wildcard matching typically provide the most hits at the expense of precision (a fraction of retrieved instances that are relevant). However, as they are later in the query progression sequence, they are also ranked lower than exact matches, if such exist. For instance, two examples of such fuzzy queries are: (1) “cron disease” (typo in Crohn), which returns the following top three results- “Crohn Disease”, *“*Crohn's disease”, and *“*Crohn's disease of large bowel”; and (2) search for “myleoid leukemia” (typo in myeloid), which returns “Myeloid Leukemia”, “Primary Myelofibrosis”, and “Leukemia Myelocytic Acute”.

An example of one of the more powerful features of the Oracle Text search is the ACCUMulate operator that allows parts of the query that did not match to be ignored. That means that it is not necessary to artificially restrain the number of keywords in a query. For example, searching for “cystic fibrosis gene carrier” returns “Cystic fibrosis gene carrier” (all tokens matched), “Carrier of cystic fibrosis gene mutation” (all tokens matched, “of” and “mutation” were ignored), “Encounter due to being a cystic fibrosis carrier” (only “cystic”, “fibrosis”, and “carrier” tokens matched, all others were ignored). In this case, only the first result matched the exact phrase, while the second result had all the keywords, but in a different order, and finally, the last result did not include the keyword “gene”.

### Custom Terminology Browser

The exploration of the UMLS is challenging because of its complexity and lack of obvious starting points, typical of more formal classifications. The UMLS is often displayed as a tree of high level root concepts for the underlying terminologies (cf. the NLM browser provided by the UMLS Terminology Services), but it is in fact more of a tightly interwoven mesh, as it integrates multiple ontology sources with often overlapping coverage and different layers of granularity. Previously, it has been demonstrated that the UMLS is a scale free network that contains both noisy concept hubs (that do not generate meaningful transitive connections, eg, Sudden onset, attribute) and informational concept hubs (that are indispensable for generating useful cross terminology connections, eg, Fever) [24].

Additionally, a graph of UMLS size cannot be effectively analyzed using available state of the art network analysis software, for example, Cytoscape [25]. For this reason, we hypothesized that the more often a particular concept occurs in different sources, the more relevant it is in UMLS navigation. We ranked all the UMLS concepts according to their branching factor (number of children) and number of unique source mappings. The one hundred top ranked concepts were then selected from SNOMED CT, LOINC, and RxNorm separately to achieve equal representation in the final result set of 167 nodes and 230 edges (some concepts overlapped). LOINC codes and parts were considered independently due to their different nature [15]. This smaller network was then plotted in Cytoscape using its hierarchical layout, and 19 identified root concepts formed the entry points for the default MU 360 tab in the ClinMiner terminology browser (Figure 1).

This MU 360 view is a custom UMLS perspective integrating all its sources with a specific focus on MU terminologies. For the purpose of hierarchical display and browsing, we adopted a conservative approach and limited the UMLS traversal to either the UMLS itself, or any of the following terminologies specific to MU- RxNorm, NDF-RT, LOINC, SNOMED CT, HCPCS, and ICD-9-CM. We explicitly ignore hierarchical relations from other terminologies, as in our experience they may add nonsensical paths to query expansion, for example, between “myocardial infarct” and *“*dermatologic disorders” via *“*disorder of soft tissue”. Additionally, to augment relatively flat LOINC and RxNorm hierarchies, some other relations are treated here as hierarchical, for example “class_of” and “measured_by” in the case of LOINC. This can be seen in Figure 1, where LOINC tests measuring warfarin concentration appear as children of the Warfarin concept.

An example of one of the unique features of mature terminologies such as SNOMED CT in contrast to more simple classification systems such as ICD-9, is that a single concept can exist in multiple places of the hierarchy, for example, “bronchitis” has two parent terms, “infection” and “bronchial disease”. This is difficult to display using a tree like hierarchy, as it requires multiple tree fragments. Instead, the ClinMiner terminology browser displays the active term, all of its parents, siblings, and children terms in three horizontally aligned panes at the same time. The Rat Genome Database originally introduced this approach [26]. When a term is clicked in any of the columns, it becomes the active term and moves to the center column together with its siblings, while adjacent columns update to show parent terms to the left and children terms to the right (Figure 1). This allows for easy exploration of the ontology in both directions, with three levels of terms being visible at all times, and supports multiple inheritance (multiple parents) in a single view.

### Extract, Load, and Transform

**Figure 2:**
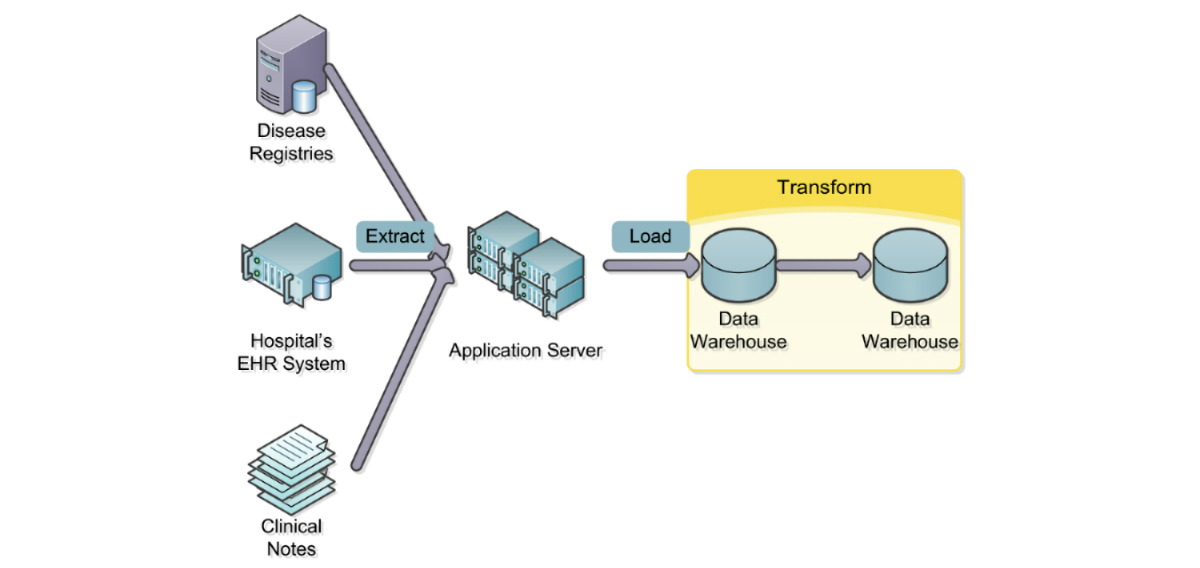
An overview of the extract, load, and transform (ELT) process. Data is extracted from multiple sources including disease registries, hospital’s EHR system, and clinical notes.

**Figure 3:**
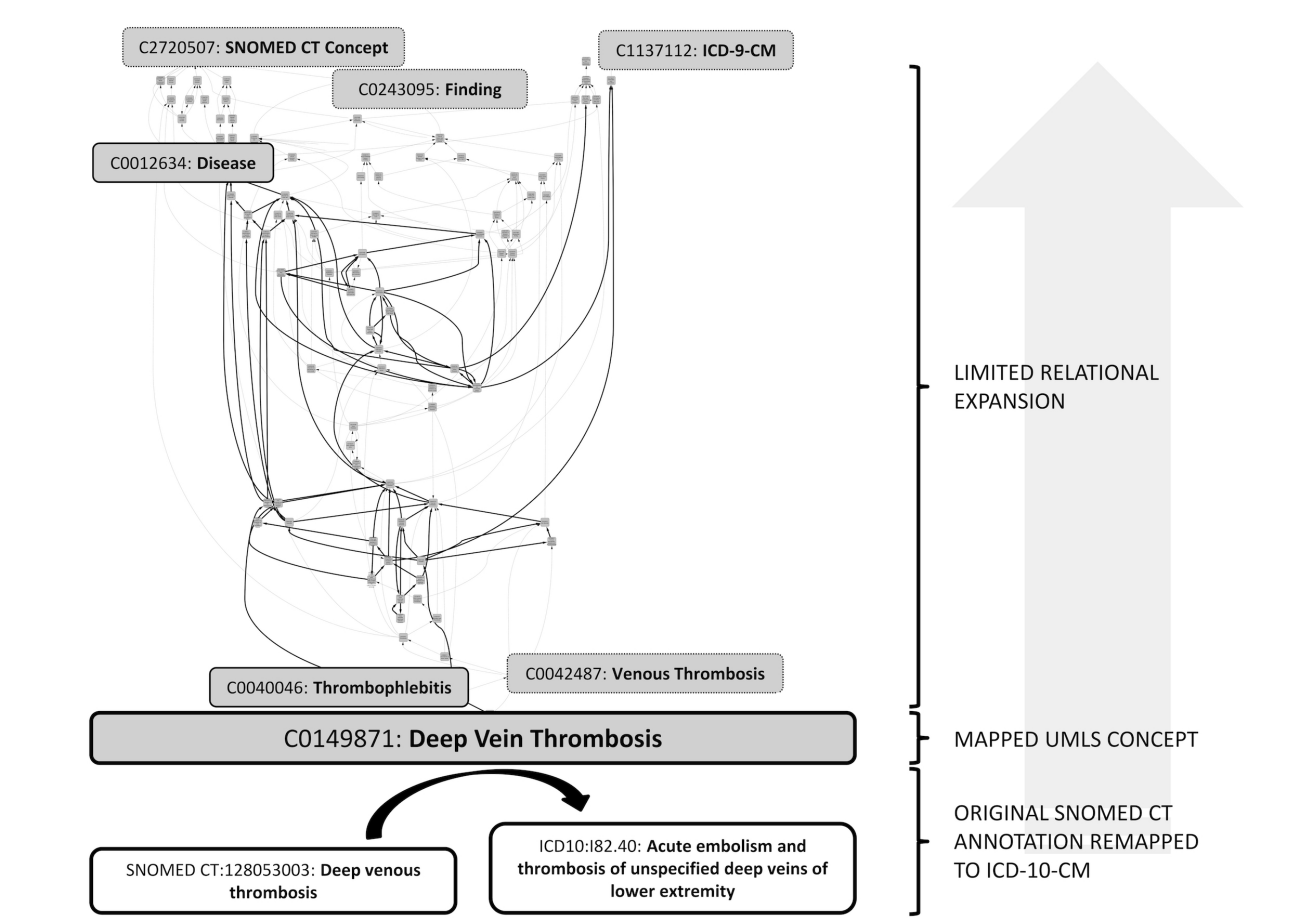
An example of the transformation stage in the extract, load, and transform (ELT) process (for a higher resolution image, see Multimedia Appendix 1). The SNOMED CT annotation "Deep venous thrombosis" made originally in the EHR, is mapped in the UMLS to the "Deep Vein Thrombosis" concept, and can be further remapped into the UMLS source concepts such as the ICD-10-CM "Acute embolism and thrombosis of unspecified deep veins of lower extremity" concept shown in the lower right portion of the figure. The UMLS concept "Deep Vein Thrombosis" is then expanded across a set of parent concepts that are within the same UMLS Semantic Network (solid lines). The concepts characterized by a different semantic type are not included in the expansion (dotted lines). In this example, two parent concepts of "Deep Vein Thrombosis", "Thrombophlebitis and Venous Thrombosis" have semantic types "Disease" or "Syndrome and Pathologic Function" respectively. Thus, the expansion does not include the term "Venous Thrombosis", as the semantic type is different from the originating concept’s semantic type ("Disease or Syndrome"), but does include "Thrombophlebitis", which share the same semantic type. There were four high level concepts that were additionally highlighted at the top of the figure, out of which "Disease" is the only one included in the expansion.

An automatic process translates original codes in patients’ EHR data to their corresponding UMLS concept unique identifiers (CUIs). Figure 2 illustrates an overview of this, and Figure 3 shows the details of the transformation. This is a bidirectional process, as the UMLS codes are also projected back into source terminologies, which can reactivate concepts (when the UMLS CUI is mapped to both active and retired versions of the same concept in the source vocabulary), as well as provide views based on terminologies that were not originally used to annotate the data. For example, in the demonstration it is possible to explore the Clinical Avatars data in the NCI Thesaurus and OMIM tabs (Figure 1), although no direct mappings to the NCI Thesaurus or OMIM were made initially. This is also illustrated in Figure 3, where the original SNOMED CT concept “Deep venous thrombosis” (SNOMED CT:128053003) is translated via the mapped UMLS concept “Deep Vein Thrombosis” (C0149871) into the ICD-10-CM concept “Acute embolism and thrombosis of unspecified deep veins of lower extremity” (ICD10:I82.40). To see the contents of each individual node in this transformation, please see Multimedia Appendix 1.

To facilitate queries across thousands of patients, the transformation process also includes query expansion and complement creation. For example, a patient with “deep vein thrombosis” and “acute myocardial infarction” would, at this step, also be automatically annotated with “cardiovascular diseases”, the parent term for these two concepts, as well as negated “No past history of venous thrombosis” and “Myocardial perfusion normal”, when no annotations were made to these concepts for this patient. In addition to the intentional restrains on the UMLS traversal described earlier, query expansion is limited to concepts that are within the same UMLS Semantic Network (ie, sharing the same semantic type), as shown in Figure 3.

In terminologies that use multiple inheritance as a design pattern (eg, SNOMED CT vs ICD-9), a single term can exist in multiple paths. Additionally, different granularities and overlap across source terminologies lead to hierarchical cycles (loops). Patient level query expansion adds to this complexity as patients can have multiple annotations of the same type or varying levels of overlapping granularity (see the earlier example of “bronchitis” and “infection”). The simple addition of branch counts would in this case lead to inflated numbers. For this reason, sets of unique patient identifiers have to be propagated across the ontology graph to precalculate accurate patient level counts at every level of the ontological hierarchy, which would eliminate the aforementioned issues and produces a directed acyclic graph. From this, it is straightforward to calculate the propagated negated information as a relative complement of a set of propagated patient terms with respect to all propagated terms across all patients.

In order to minimize the user effort involved in browsing large hierarchies, the ontology graph is additionally approximated as a minimum Steiner tree problem [27]. This produces a more compact reconnected terminology tree, which includes only the concepts that appear in the selected dataset and their best connected parent concepts, rather than all of the potentially available concepts within the UMLS graph. Selecting from the “Data-driven perspective” drop-down in Figure 1 enables this view. This process also identifies orphaned nodes that were otherwise disconnected from hierarchy, placing them at the root of the tree.

### Web Front-End

The application was developed in Java using enterprise Java technologies- Spring Framework, Spring Roo, Java Persistence Application Programming Interface (Java Persistence Application Programming Interface, EclipseLink provider), Apache Maven, and AspectJ Vaadin, a Java Web application framework that extends Google Web Toolkit, was used to provide the rich Internet application experience. Apache Tomcat provided the Web container. The Apache Hypertext Transfer Protocol Server isolates the Web container and forces encryption on all browser connections with 256-bit Transport Layer Security.

### Data Sharing

Requests for a virtual machine image containing a preconfigured version of ClinMiner can be made to the corresponding author. We also welcome data submissions to our local instance, which can then be securely accessed over the Internet, so there is no need for additional deployment.

## Results

### Simulated Data

To drive the initial implementation, we used simulated patient data kindly provided by the Clinical Avatars project [28]. The Laboratory for Personalized Medicine created the Clinical Avatars and developed a methodology for creating virtual representations of people for the purpose of conducting personalized medicine simulations. This simulation uses a realistic statistical distribution of patient characteristics such as age, gender, ethnicity, and genotype based warfarin response, and represents a typical set of elements that a researcher would expect in a clinical trial. All avatars data included genotype information on two genes important in warfarin pharmacogenetics: (1) CYP2C9, warfarin metabolizing enzyme; and (2) VKORC1, Vitamin K epOxide Reductase Complex 1. The polymorphisms in these genes are clinically important, as they affect therapeutic warfarin ranges across different racial groups [29]. In this particular case, the dataset used represented a simulation of 100,000 patients (10,836,196 observations) undergoing genotype guided warfarin dosing in the process of initiating oral anticoagulation over 90 days using the Couma Gen protocol [30].

The Clinical Avatars data elements were manually mapped using MU ontologies. The mappings were than validated, and the final set is shown in Table 2. A similar approach is used when annotating real clinical notes, and for this purpose we developed and maintain an internal standard operating procedure. The EHR data has an additional extraction step, where a custom parser strips irrelevant information and encounter based data is transformed into time stamped observations. All preexisting codes in the EHR are loaded *as is*.

### Electronic Health Records Data

A “Limited Dataset”, as defined under the Health Insurance Portability and Accountability Act, encompassing 7931 patients was obtained from the Medical College of Wisconsin Clinical Research Data Warehouse for this study. The data extract was in the form of standard Epic Clarity tables for a subset of patients that had an encounter or a problem list in the “Malignant neoplasm of pancreas” (ICD9:157) or “Epilepsy and recurrent seizures” (ICD9:345) code subset. Epic Clarity is an SQL relational database extracted for reporting purposes from Epic Chronicles, the data engine at the heart of Epic’s EHR.

The drug information in the EHR was encoded using Medi-Span terminology, one of the RxNorm sources, which facilitated its automatic translation into the UMLS. The clinical results were encoded as orders using CPT-4 codes or using a fixed category from the “CLARITY_COMPONENT” lookup table. We have manually mapped the top 130 most frequently performed laboratory tests (out of a total of 7766 records in the EPIC “CLARITY_COMPONENT” table) to LOINC, which provided coverage for 94.07% (4,765,012/5,065,315) of all the laboratory tests. The remaining 5.93% (300,303/5,065,315) laboratory tests were left unmapped.

A practical difference between simulated and real EHR data is the much larger concept space, which in this case covered 13,614 unique ICD-9, CPT-4, LOINC, and RxNorm codes. This code set was remapped into the UMLS, which resulted in 13,383 distinct UMLS CUIs, and then expanded as described previously across a limited set of “is_a” and selected other relationships (eg, “has_ingredient”) to facilitate querying, which produced the final set of 30,153 concepts. We have successfully applied this approach in a separate study focused on association rule mining in pancreatic cancer [31].

A demonstration limited to Clinical Avatars data is available on the Internet using the credentials user “jmirdemo” and password “jmirdemo” [32].

## Discussion

### Extract, Load, and Transform

The crosswalk via the UMLS between different terminologies, as demonstrated in Figure 3, is important for several reasons. Where records are coming from legacy sources, they may use an older coding scheme, for example, ICD-9 or NDC, and this process makes the data browsable via a more expressive terminology, such as SNOMED CT. Additionally, the UMLS transformation alleviates the issue of variability in coding across data sources that use different terminologies, for example, drug information annotated with the Veterans Health Administration National Drug File - Reference Terminology and Medi-Span Master Drug Data Base terminologies can both be reconciled using RxNorm.

The extract, load, and transform approach is substantially different from a more common extract, transform, and load approach, when data is transformed before it is loaded into the data warehouse. Conversely, with extract, load, and transform, we essentially maintain two versions of data: (1) the original annotation set made in the EHR, and (2) a dynamically generated set of UMLS mappings. The original data is never lost, and can be retransformed as new knowledge becomes available.

We are now working on expanding the transformed information to include date and values to support more advanced temporal and value restricted queries. This is a critical step that has a significant impact on the time required to query patient information, however, the actual transformation is relatively resource consuming, for example, it creates 238 annotations per avatar using simulated data and several thousand annotations per patient with real EHR data.

### Search and Complexity

A relatively large number of concepts remain unused when annotating clinical data to large terminologies. The UMLS, the largest repository of biomedical terminologies, in its current version spans over 10 million unique concept names from over 160 source vocabularies. Only a subset of the UMLS might be suitable for concept matching [33], and SNOMED CT alone may be enough to represent most of the terms commonly used in medical problem lists [34]. In this study, a cohort of eight thousand patients required between ten and thirty thousand (with query expansion) concepts to capture all clinically relevant features.

While physicians rarely have to deal with ontology hierarchies directly, these are indispensable in clinical research to facilitate query expansion, building transitive closures, and data validation and reconciliation. Any sufficiently large terminology is likely to suffer from some inconsistencies and these, however minor, present unique challenges for ontology end users when they have no direct control over the terminologies they are using. With hundreds of thousands of concepts, traditional navigation through terminology hierarchies becomes impractical. This is why we put a special focus on enhancing search capabilities as well as providing data-driven perspectives that dynamically hide some of this complexity. The search becomes even more important when concepts do not appear where expected or are not in hierarchical relations at all. In our experience, this is the case in approximately one third (data not shown) of LOINC and RxNorm concepts.

### Beyond Meaningful Use

Current requirements for terminology standards are not necessarily intuitive and are likely to cause confusion among implementers and subsequent interoperability issues. Optionality for some of the vocabulary standards only adds to the confusion. Existing studies suggest that there is a wide variation in accuracy of MU electronic reporting [35]. Even within a single terminology providers can significantly differ in which code they assign to the same observation [36]. While there are numerous challenges to data capture, the community can best address them through standardization and convergence on key data elements [37].

Interestingly, several resources in the genotype to phenotype space now actively map to the UMLS directly: (1) Orphanet, a portal for rare diseases [38]; (2) the Human Phenotype Ontology project, which provides a structured description of human phenotypic abnormalities [39]; and (3) ClinVar, a novel National Center for Biotechnology Information database for clinical genomics [40], are all good examples of resources that rely on the UMLS to integrate clinical features, conditions, genes, and proteins.

The UMLS incorporates decades of experience and consistency represented by the US National Library of Medicine, which in fact already maintains RxNorm, one of the MU terminologies. It is therefore not unfeasible that the UMLS could provide a clearer path to semantic interoperability.

### Conclusions

Despite its inherent complexity, the UMLS can serve as an effective interface terminology for many of the clinical data standards currently used in the health care domain.

## Abbreviations

CDT: Code on Dental Procedures and Nomenclature
CMS: Centers for Medicare & Medicaid Services
CMT: Convergent Medical Terminology
CPT: Current Procedural Terminology
CUI: concept unique identifier
EHR: electronic health record
HCPCS: Health care Common Procedure Coding System
ICD-9-CM: International Classification of Diseases Ninth Revision Clinical Modification
ICD-10-CM: International Classification of Diseases Tenth Revision Clinical Modification
ICD 10 PCS: International Classification of Diseases-10 Procedure Coding System
KP: Kaiser Permanente
LOINC: Logical Observation Identifiers Names and Codes
MU: meaningful use
NDC: National Drug Code
NLM: National Library of Medicine
OMB: Office of Management and Budget
OMIM: Online Mendelian Inheritance in Man
SNOMED CT: Systematized Nomenclature of Medicine, Clinical Terms
SQL: Structured Query Language
UMLS: Unified Medical Language System

## Multimedia Appendix 1

An example of the transformation stage in the extract, load, and transform (ELT) process (a version of Figure 3 with higher resolution). The contents from each individual node can be viewed here.
